# Ambient air pollution and thrombosis

**DOI:** 10.1186/s12989-017-0237-x

**Published:** 2018-01-03

**Authors:** Sarah Robertson, Mark R. Miller

**Affiliations:** 1 Centre for Radiation, Chemical and Environmental Hazards, Public Health England, Harwell Science and Innovation Campus, Didcot, Oxfordshire OX11 0RQ UK; 2 University/BHF Centre of Cardiovascular Science, University of Edinburgh, Edinburgh, UK

**Keywords:** Air pollution, Particulate matter, Ozone, Nitrogen dioxide, Diesel exhaust, Thrombosis, Coagulation

## Abstract

Air pollution is a growing public health concern of global significance. Acute and chronic exposure is known to impair cardiovascular function, exacerbate disease and increase cardiovascular mortality. Several plausible biological mechanisms have been proposed for these associations, however, at present, the pathways are incomplete. A seminal review by the American Heart Association (2010) concluded that the thrombotic effects of particulate air pollution likely contributed to their effects on cardiovascular mortality and morbidity. The aim of the current review is to appraise the newly accumulated scientific evidence (2009–2016) on contribution of haemostasis and thrombosis towards cardiovascular disease induced by exposure to both particulate and gaseous pollutants.

Seventy four publications were reviewed in-depth. The weight of evidence suggests that acute exposure to fine particulate matter (PM_2.5_) induces a shift in the haemostatic balance towards a pro-thrombotic/pro-coagulative state. Insufficient data was available to ascertain if a similar relationship exists for gaseous pollutants, and very few studies have addressed long-term exposure to ambient air pollution. Platelet activation, oxidative stress, interplay between interleukin-6 and tissue factor, all appear to be potentially important mechanisms in pollution-mediated thrombosis, together with an emerging role for circulating microvesicles and epigenetic changes.

Overall, the recent literature supports, and arguably strengthens, the contention that air pollution contributes to cardiovascular morbidity by promoting haemostasis. The volume and diversity of the evidence highlights the complexity of the pathophysiologic mechanisms by which air pollution promotes thrombosis; multiple pathways are plausible and it is most likely they act in concert. Future research should address the role gaseous pollutants play in the cardiovascular effects of air pollution mixture and direct comparison of potentially susceptible groups to healthy individuals.

## Background

Outdoor air pollution is estimated to be responsible for over 3 million premature deaths worldwide [1], and thus represents one of the leading risk factors for all cause disease [2, 3]. Of these, the vast majority of deaths are attributed to cardiovascular disease (CVD) [1]. Thrombosis is the most common underlying pathology triggering the two major cardiovascular disorders: coronary heart disease and stroke. While many of the biological mechanisms underlying the link between air pollution and CVD remains uncertain, the seminal American Heart Association (AHA) review concluded that thrombotic mechanisms may in part explain the observation that exposure to air pollution is associated with adverse cardiovascular events [4]. The aim of the current review is to examine the newly accumulated scientific evidence (2009–2916) on whether or not mechanisms of haemostasis and thrombosis contributes towards CVD induced by exposure to air pollution. The remit of this review also extends to gaseous pollutants, which had not been previously reviewed by the AHA statement [4]. This work forms part of a larger ongoing piece of work being undertaken by the Committee on the Medical Effects of Air Pollutants on estimating the effect of long-term exposure to ambient air pollution on cardiovascular morbidity in the UK today

Air pollution is a complex heterogeneous mixture of gases and particles, arising from a wide variety of stationary and mobile sources; both directly emitted (primary emissions) or formed within the atmosphere (secondary emissions). From a health perspective, nitrogen oxides (NOx) and particulate matter (PM) currently receive the greatest attention, although ozone (O_3_) and sulphur dioxide (SO_2_) also have potential to cause harm. PM is itself a complex mixture of airborne particles that differ in size, origin and chemical composition. Particles are classified into three classifications based on aerodynamic diameter; coarse (PM_10_, 2.5–10 μm) fine (PM_2.5_, <2.5 μm) and ultrafine (UFP, <100 nm). At present only PM_10_ and PM_2.5_ are widely monitored (and regulated) in the environment, with most attention given to PM_2.5_ due to the greater penetration into the lung alveoli and its high reactive surface area for a given mass. On this basis and others (e.g. translocation into the blood, different surface composition), UFPs could represent a greater threat to health, but at present cannot be measured routinely in large numbers of individuals without using surrogates such as particle number count (PNC).

**Fig. 1:**
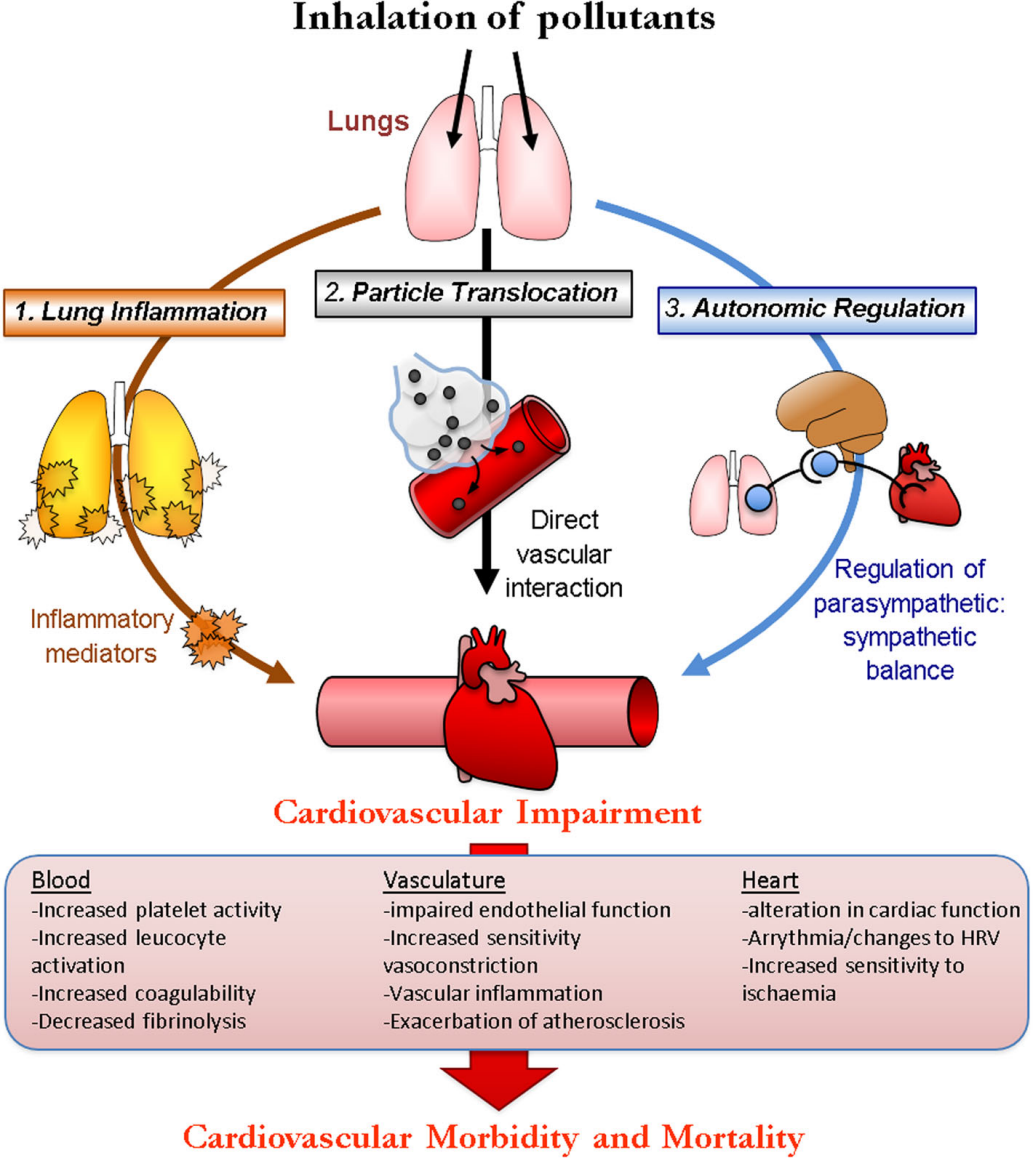
Flowchart showing the three main hypotheses of how inhaled particles could cause cardiovascular impairment. Adapted from Niemann et al. [78]

There is persuasive evidence, particularly for PM, on the negative impact of air pollution on cardiovascular events and outcomes, including electrocardiographic changes (e.g. reduced heart rate variability), endothelial dysfunction, atherosclerosis and thrombosis. The biological mechanisms underpinning the effects of air pollution on CVD, however, remain poorly defined. Nonetheless, three main hypothesis have been proposed by which air pollution that is inhaled into the pulmonary system can then instigate remote cardiovascular effects: 1) particle induced inflammatory responses in the lungs, leading to the release of inflammatory and oxidative mediators into the circulation; 2) pollutant-induced activation of airway sensory nerves resulting in autonomic imbalance; and 3) direct entry of pollutants (usually with a focus on particles or chemical constituents) into the pulmonary circulation before being carried into the systemic circulation (Fig. 1).

**Fig. 2:**
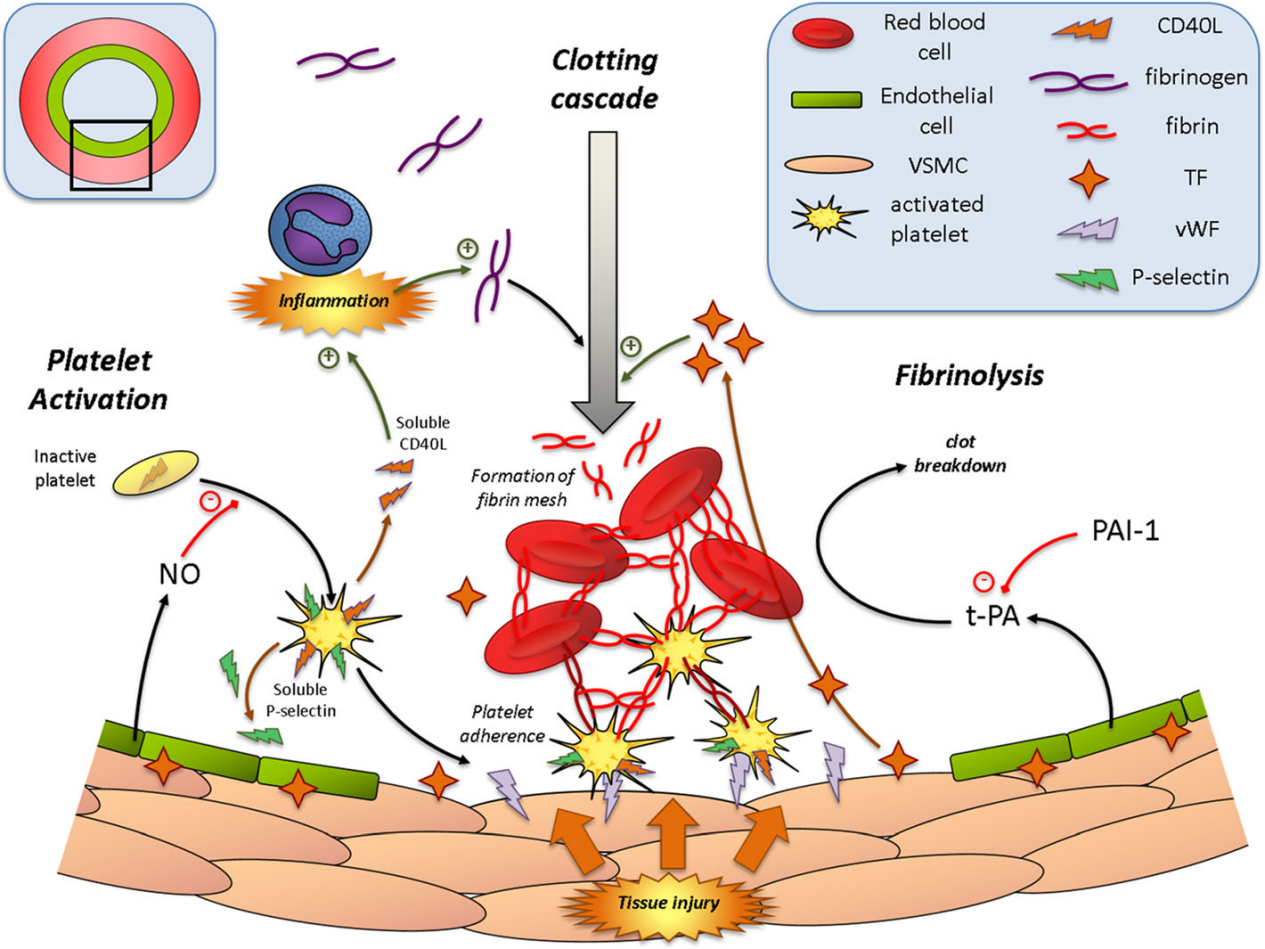
Processes and components involved in normal haemostasis (these are discussed in more detail in the text)

**Fig. 3:**
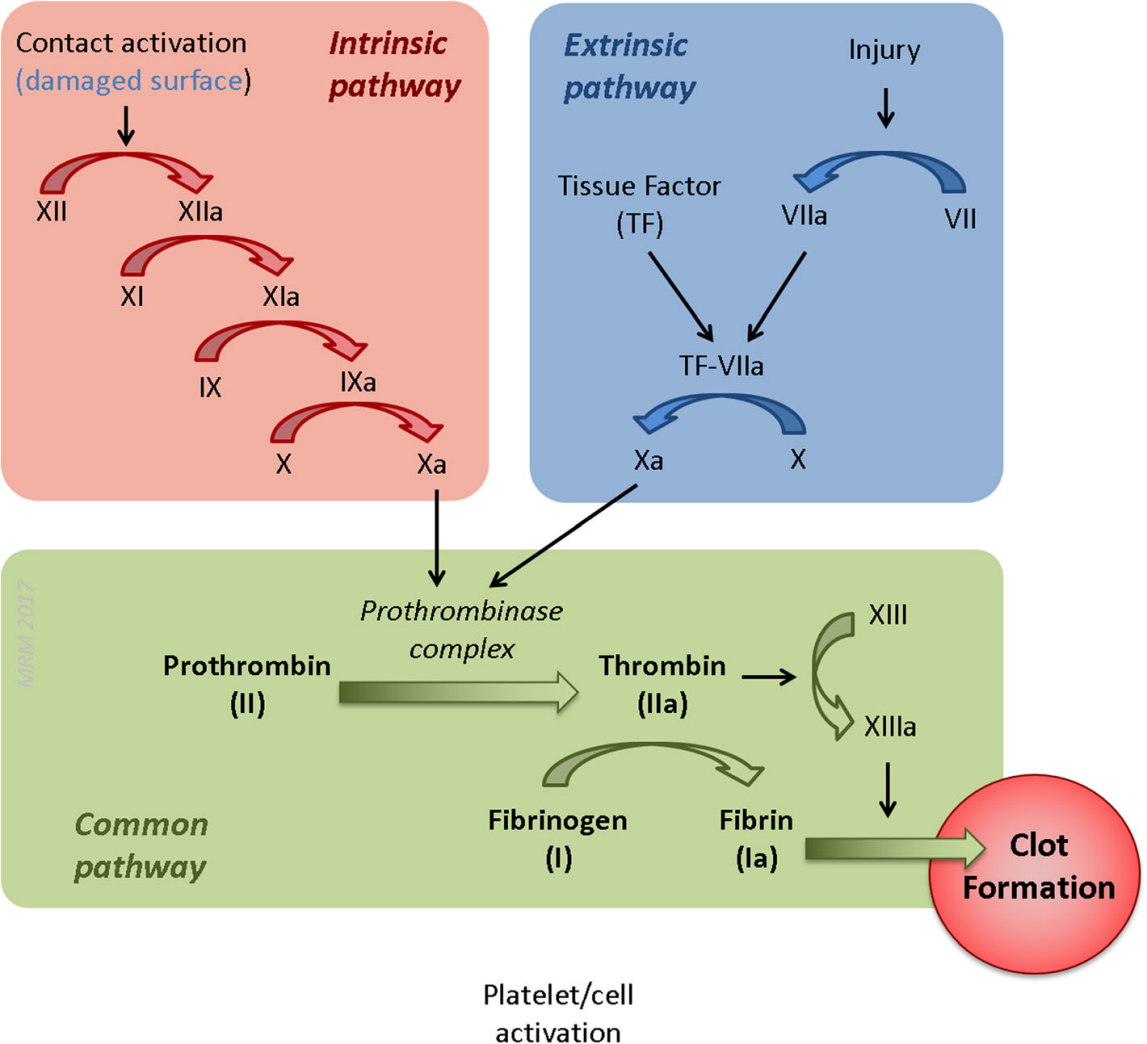
The intrinsic, extrinsic and common pathways of the coagulation (clotting) cascade. Air pollution has been shown to affect several steps in blood coagulation (these are discussed in more detail in the text)

Haemostasis is a complex, orchestrated series of event events to maintain circulating blood in the liquid state and to prevent blood loss following injury through the formation of a blood clot. Excessive clotting, especially in patients with pre-existing CVD, can block major arteries leading to a loss of downstream blood flow, and potentially leading to clinical events such as heart attack, ischaemic stroke or death. In contrast, a reduction in the ability of blood to clot can lead to uncontrolled bleeding with severe blood loss from injury or escape of blood following aneurysm, e.g. thrombotic stroke. Thus the body maintains an intricate balance to preserve haemostasis; a process that involves the interplay of circulating blood cells, a variety of coagulation factors, platelets and fibrinolytic factors, as well as interactions with the vascular wall and endothelial cell-derived mediators (Fig. 2). Clotting may be initiated by either the intrinsic (contact activation) or extrinsic pathway (tissue factor; TF). These two pathways converge into a final common pathway of thrombin production and fibrin clot formation (Fig. 3). In this review, we use the term “haemostasis” to include the whole process: platelet activation, coagulation and fibrinolysis. Exposure to air pollution has been shown to influence each of these dynamic processes, with increasing evidence suggesting that the overall haemostatic balance is shifted towards a pro-coagulant and anti-fibrinolytic state [4]. The 2010 AHA report [4] concluded that there was evidence, albeit somewhat inconsistent, suggesting that PM may adversely affect haemostasis shifting the balance to a pro-coagulant and anti-fibrinolytic state and that this may, in part, contribute to the effects of air pollution on CVD. The mechanisms underlying such an effect were poorly defined but systemic inflammatory activation and alterations in platelet function are proposed as key processes involved in the alterations in haemostasis [4].

This review provides an overview of the updated literature in the area of air pollution, which has growing increasingly topical, with a specific focus on the thrombotic actions given the contribution that pathway could make to the substantial cardiovascular morbidity of air pollution. In our overview we highlight recent advances and explore mechanistic understanding of the pathways linking air pollution exposure and haemostasis parameters. Improved understanding is needed to provide crucial insight into which pollutants are most harmful, who may be most at risk, and informs future research needs and public health policy in respect of advice and interventions.

## Search strategy and review structure

### Search strategy

This work uses the 2010 AHA scientific statement [4], as an expert review of the literature between January 2004 to March 2009, as a foundation for reviewing the research in this area in the subsequent years. Literature searches were performed in PubMed from the dates of 1st January 2009 to 28th February 2016, using the following search terms: “air pollution” or “particulate matter” *and* “blood” or “thrombosis” or “clot” or “fibrinolysis” or “coagulation” or “embolism” or “platelet”. Terms for gaseous were not included due to the large number of irrelevant references these terms produced. Preliminary checks were performed to ensure that relevant references with gaseous pollutants were captured by the term “air pollution”. Other papers were identified from prior knowledge, contact with experts in the field and hand searching of the bibliographies of the papers identified in the electronic search. References were downloaded into the referencing software program Endnote (version X8).

### Study selection

**Fig. 4:**
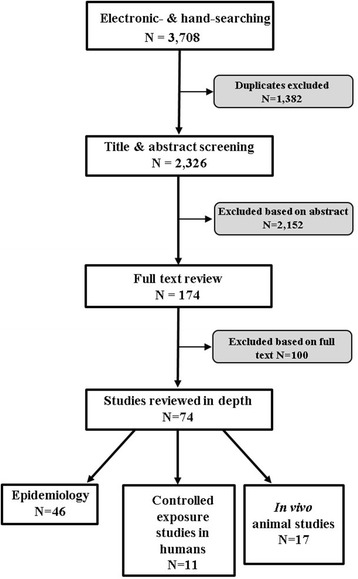
Flowchart showing the numbers of papers included/excluded at each stage of the search strategy

A total of 2326 publications were identified following removal of duplicates (Fig. 4). These were screened first at the abstract level, and then at the full article level. To be included in the final analysis studies had to meet the following inclusion criteria:

- Peer-reviewed articles or published by a recognised institution between 1st January 2009 and 28th February 2016*
- Study type: epidemiological studies, human controlled exposures and intervention studies, in vivo (animal) studies
- Exposure: ambient (outdoor) air pollution – particulate air pollutants**, diesel exhaust (DE), O_3_, nitrogen dioxide (NO_2_), carbon monoxide (CO), SO_2_
- Health outcome: reported on coagulation, fibrinolysis, thrombophilia and platelet profile
- Population: general population (all ages, those with pre-existing health conditions)

* Key earlier publications are discussed for contextual background.

** Included PM size fractions, black carbon (BC), concentrated ambient particles (CAPs) and PM from DE (i.e. diesel exhaust particles (DEP)).

Key exclusion criteria included:

- No original data included (e.g. reviews, editorials and commentaries were excluded). However, reference lists from the identified original articles and reviews were screened to identify any other potentially relevant studies.
- Language: full text not available in the English Language
- Study type: in vitro
- Exposure not relevant to ambient air pollution (e.g. indoor air pollution, occupational exposure***, biomass, cigarette smoke, manufactured nanoparticles)
- Did not provide any mechanistic data beyond mortality or hospital admission

*** Occupational exposure to manufactured nanoparticles, industrial accidents and environmental events (e.g. volcanic eruptions or wildfires) were not included. Workplace exposures that potentially spill into communities or were representatives of the main aspects of general urban air pollution (e.g. engine emissions from garages, bus depts.) were included.

### Review structure

This review provides a narrative summary of the seventy four publications that fulfilled all inclusion criteria. The piece has been structured by study type: epidemiological studies (62%) controlled exposure studies in man (15%) and in vivo animal studies (23%). Epidemiological studies were further subdivided into short-term (7 days of exposure or less) versus long-term (>1 week of exposure). Emphasis has been given as to whether the conclusions of the AHA statement have been strengthened during the 8-year period and where new insight has been made as to possible underlying mechanisms. Of note, we expand the remit from the AHA statement by inclusion of gaseous pollutants (e.g. NO_2_, O_3_, CO and SO_2_).

## Epidemiological studies

### Short-term exposure to air pollutants

The vast majority of epidemiological studies addressing the haemostatic effects of air pollution have investigated acute exposures (<7 days), with most evaluating alterations in plasma biomarkers of systemic coagulation and fibrinolysis. Fibrinogen, an essential coagulation protein associated with an increased risk of coronary events [5, 6], has been one of the most studied. Hildebrandt and colleagues reported PM to be associated with a 2.4% increase in fibrinogen from 3.1 g/L (baseline) in 38 male patients with chronic obstructive pulmonary disease (COPD) [7]. However, no clear pattern emerged for the other plasma haemostatic biomarkers evaluated. Thus, the increase in fibrinogen in response to exposure may be somewhat artificial, perhaps arising from small sample bias. With a larger sample size (*N* = 242), another study noted no increase plasma fibrinogen levels in patients with COPD per interquartile range (IQR) increase in PM_2.5_ at all lag days (0–10) [8]. Similarly, there were no significant changes in fibrinogen with PM exposure among young and older adults [9–11]. Besides fibrinogen, other biomarkers having pro-coagulant properties or reflecting a pro-thrombotic state have been investigated (including, (plasminogen activator inhibitor type 1 (PAI-1) and tissue-type plasminogen activator (t-PA)) [10, 12, 13]. Earlier studies typically analysed single biomarkers of haemostasis, whereas more recent studies have used a multiple biomarker approach. While most studies have observed pollution-related changes in at least one biomarker, the direction and statistical significance of the pollutant-biomarker association has not been consistent, making it difficult to generalise conclusions. More convincing evidence for associations between exposure to ambient particles and biomarkers of haemostasis comes from studies conducted during the 2008 Beijing Olympics [10, 14]. The air pollution control measures that were put in place in Beijing during the 2008 Olympic Games provided an unprecedented opportunity for quasi-experimental studies to assess the effect on systemic biomarkers of haemostasis and inflammation, among others. The short-term reductions in PM during the Olympic Games was associated with decreases in biomarkers of haemostasis [10, 14]. During the 2008 Beijing Olympic Games, Rich et al. (2012) observed statistically significant improvements in soluble P-selectin (sP-selectin; −34%) and von Willebrand (vWF; −13.1%) among, the 125 healthy young adults studied [14]. An interesting study by Delfino et al. (2009) examined whether medication use modifies the association between particulate air pollution and biomarkers of haemostasis. Here, an association between PM_2.5_ exposure and blood levels of sP-selectin in coronary artery disease (CAD) was only observed in those not taking clopidogrel, providing further evidence for a role of platelets [12]. Further studies are warranted to disentangle the modifying effects of health status and use of different medications, in addition to whether such preventative action of anti-platelet agents are more marked in those with greatest exposure to air pollutants.

Inconsistencies between studies are also likely to reflect the differences in the accuracy of the exposure assessment for individual volunteers. Studies have generally relied on data collected from the nearest fixed-ambient monitoring stations as a surrogate for personal exposure. Unless complex modelling has been performed a key weakness in using fixed-site monitoring data is that it ignores spatial variability. Differences in particle composition across study locations could also be a reason for the inconsistent findings, as physical and chemical characteristics of PM are known to vary widely both with space and time [15, 16]. Very few studies have analysed associations between individual PM constituents and changes in biomarkers of haemostasis. There is limited/suggestive epidemiological evidence that the haemostatic responses are more intrinsically related to chemical composition rather than just particle mass. For example, Wu et al. (2012) demonstrated that certain transition metals (e.g. iron, titanium, cobalt and cadmium) within PM_2.5_, but not PM mass, was associated with increased plasma fibrinogen levels among healthy adults in Beijing. Besides fibrinogen, biomarkers of the fibrinolytic pathway (PAI-1 and t-PA) were also significantly and positively associated with transition metals in PM_2.5_. These epidemiological observations are consistent with animal and in vitro studies showing a role for transition metals in the PM-induced toxicity [17]. Additionally, studies have shown the administration of chelating agents (chemical compounds that react with metal ions, to decrease their availability for other reactions) can block the effects of air pollution on blood coagulation [18]. Several transition metals have been shown to participate in Fenton and Fenton-like reactions and thus induce oxidative stress. Oxidative stress is a reoccurring mechanism in actions of air pollution [19], and associations between exposure and biomarkers of oxidative stress have been observed in both in vivo and in vitro studies [20]. To the best of our knowledge, only one study that has investigated the effects of genetic polymorphisms related to oxidative stress and particulate pollution exposure on markers of haemostasis [21]. Stronger associations between PM and haemostatic biomarkers were observed among individuals who were genetically predisposed to oxidative damage [21]. However, unexpectedly, exposure was associated with a decrease in the levels of circulating biomarkers of haemostasis, counter intuitively suggesting a potentially protective effect of PM against thrombosis [21]. Besides oxidative-stress related polymorphisms, other epidemiological studies have investigated the effect of polymorphisms in the fibrinogen genes on responses to PM exposure [22]. For example, carriers of the FGB rs1800790 minor allele elicited greater fibrinogen responses to PM than those with a major allele [22]. Such ‘unforeseen’ functional consequences of genetic polymorphisms may, in part, account for discrepant findings between studies investigating different populations. Further work in this area is clearly warranted.

Size distribution in ambient air may also be important. An increasing body of experimental data (see below) has shown that the ultrafine fraction within PM is more toxic than the fine or coarse fractions, attributed to the larger surface area per unit mass of ultrafine particles (UFPs). The large majority of these studies have focussed on pulmonary responses and as yet, there are too few population studies to conclude whether different particle size fractions have different magnitudes of association with haemostatic outcomes. Only a few studies have specifically investigated the effect of particle size on the haemostatic effects of PM and results have been mixed [10, 12, 21, 23, 24]. This is likely the consequence of limited statistical power of studies together with methodological heterogeneity. Of particular interest is the study by Rückerl et al. (2014) which used a particle counter with a lower particle cut-off size than the more conventional methods. Nevertheless, analysis of the different size fractions of UFP (3–10 nm, 10–30 nm, 30–50 nm, 50-100 nm) did not reveal a certain size range to be more influential than others. Also, associations were stronger for PM_2.5_ than for UFPs; inconsistent with the notion that UFPs have greater biological activity, although it is noteworthy that this study found a reduced thrombus formation in association with PM.

There is some limited evidence that different particle sizes have different intervals of time (lag) between exposure and biomarker changes, suggesting that the mode of action may be different for the various particle sizes [10]. It has been suggested that inhaled UFPs may translocate into the circulation [25–27] with the potential for direct pro-aggregatory effects on platelets. However, particle translocation remains controversial and a recent study showed that the translocated fraction is less than 1% (mass) of the delivered dose to the lung [28]. The exact pathway for the translocation into the circulation also remains unclear. While some studies [10], report associations between haemostatic biomarkers and concentrations of ambient UFPs for time lags representing direct effects (ie. lag 0), the time course of others [29] is more likely to represent indirect effects, with findings very rarely being consistent across studies for the same biomarker-pollutant pair. It is also not clear whether inflammatory processes mediate the exposure-response relationship. It has been shown that exposure to particulate air pollution can induce platelet aggregation and increased thrombin generation without inducing significant levels of inflammation [29]. Others have suggested that an inflammatory response is an essential component in the haematological changes associated with short-term exposure to air pollution [10, 13, 30]. Of note, most studies have looked at a limited number of inflammatory biomarkers and these may not necessary encompass all aspects of the inflammation response.

There has been limited progress in the use of global coagulation tests in epidemiological studies of short-term exposure. Measurement of individual biomarkers provides only ‘snapshots’ of the overall coagulation process, and global coagulation tests can provide a more complete picture of haemostatic status. The prothrombin time (PT) test (measures activity of the extrinsic and common pathways) and the activated partial thromboplastin time (APTT) test (measures activity of the intrinsic and common pathways) are widely used global screening tests for blood coagulability. To date, efforts have primarily focused on PM. Shortened PT (i.e. greater coagulability of the blood) has been demonstrated in 1218 normal subjects from the Lombardia Region, Italy after short-term exposure to PM_10_ [31]. No changes in APTT were noted, perhaps instead pointing towards TF-dependent changes in thrombin generation. This result is contrary to a more recent study showing prolonged clotting times with increased levels of PM_10_ in a group of 233 patients with diabetes [13]. However, reduced clotting times were associated with increasing PM_10_ concentrations of the sub-acute exposure windows (day 0 to day 3) [13].

One of the most exciting areas to have developed in recent years, has been increasing interest in uncovering whether environmental exposures can impact on health through epigenetic mechanisms (alterations in gene expression and function without changing the underlying DNA sequence), such as DNA methylation, histone modifications and non-coding RNA expression [32]. Alterations of DNA methylation have been linked to various human diseases, and epidemiological studies have shown distinct DNA methylation abnormalities with exposure to air pollutants [33, 34]. Evidence for associations between (i) air pollution and epigenetic alterations and (ii) between disease states (including CVD) and epigenetic changes, lends biological plausibility for epigenetic change underpinning the effects of air pollution on haemostasis. The most compelling evidence comes from a recent study demonstrating that exposure to black carbon (BC) particles was associated with a decrease in DNA methylation (hypomethylation) of the tissue factor (F3) gene and subsequent increases in fibrinogen protein expression [35]. The specific mechanism of how air pollution exposure may alter DNA methylation has not been elucidated at present.

Unlike PM, there have been relatively few studies evaluating the relationship between gaseous pollutants and biomarkers of haemostasis, and the evidence has been mixed. The evidence has been mixed with some showing positive associations, some showing no association and a few showing inverse associations [7, 8, 11, 12, 14, 24, 29]. The heterogeneity of these studies makes the data difficult to interpret as studies have tended to use different exposures (NO_2_, CO), target populations or outcome measures.

### Long-term exposure to air pollutants

As with the studies of short-term exposure to PM discussed above, fibrinogen has been the most widely studied biomarker in long-term exposure (>1 week) epidemiological studies. Using data from the Heinz Nixdorf Recall Study (a prospective population-based cohort of 4814 German adults) plasma fibrinogen levels were found to be elevated by 3.9% among men, but not women, for each 3.91 μg/m^3^ increase in annual average PM_2.5_ [36]. This was found to be independent of short-term changes in air pollution. However, a comparative study using a higher spatial resolution (1 km as compared to 5 km) did not confirm this finding [37]. Large population-based cohort studies in the US and UK have also reported no association between fibrinogen and PM [30, 38–40]. Studies also varied in their ability to adjust for potential confounding factors (medication use, co-morbidities, socio-economic status (SES)). Additionally, different exposure patterns and sources are likely to contribute to the inconsistent results among studies. Evidence from a multi-center and meta-analysis found that long-term exposure to zinc within PM_2.5_, but not other constituents (e.g. iron and nickel) increased fibrinogen concentrations [41]. These findings add to the growing evidence that mass alone does not fully capture the toxicity potential of PM.

A few studies have looked at other markers of clotting and fibrinolysis, with considerable differences in the magnitude and direction of responses obtained [30, 37, 42, 43]. Factor VII (FVII) plays a central role in initiating the process of coagulation. A cohort study of 2086 mid-life women found an inverse relationship with FVII (−3.6%; 95% Cl, −7.8 to 0.8%) for each additional 10 μg/m^3^ of annual PM_2.5_ pollution [42], pointing towards a more hypocoagulable state. However, the measurement and analysis of one or more coagulation biomarkers may not provide a complete picture of the balance between thrombosis and lysis. For example, long-term exposure to PM led to enhanced thrombin generation in patients with diabetes, in the absence of pro-coagulant changes in coagulation parameters (FVII, FVIII, FXII) [13]. The evidence has, for the most part, been directed at particulate air pollutants. Differences in the focus and design of investigations addressing gaseous air pollutants make it difficult to draw meaningful conclusions as to the long-term relationship with coagulation markers [8, 30, 40, 42, 44, 45].

A small number of studies have made use of global coagulation tests in long-term epidemiological studies. Reduced clotting times were observed in association with PM_10_ average over one year among healthy controls and patients with deep vein thrombosis [46]. Patients with diabetes showed hypercoagulability, using endogenous thrombin potential (ETP) with PM_10_ for time windows up to 6 months in patients with diabetes [13]. Interestingly, associations between PM_10_ exposure up to one month, but not longer, and thrombin generation were dependent on TF. The authors proposed microvesicles (small membrane-bound structures secreted from different cell types) to be the main source of the TF [13]. Increased levels of TF-positive microvesicles were also detected in the plasma of diabetic patients for mean PM_10_ measurement over 1 year [13].

Induction of systemic inflammation by air pollution could contribute to a pro-thrombotic state. Consistent with this notion, Viehmann et al. (2015), using data from the Heinz Nixdorf Recall Study, observed a positive correlation between C-reactive protein (CRP; a marker of systemic inflammation) and platelet count in relation to air pollution [37]. However, other studies have found no clear patterns of association between markers of haemostasis and markers of inflammation [13, 40, 44]. In the study by Emmerechts et al. (2012), a systemic inflammatory state could not explain the pro-coagulant during the longer time windows (1 month to 1 year) [13]. Only a limited number of studies have examined whether age, gender, and pre-existing co-morbidities influence the haemostatic effects of long-term air pollution exposure and results have been inconsistent. For example, Forbes et al. (2009) found no effect modification by gender for associations for PM and fibrinogen, whereas PM exposure was associated with increasing fibrinogen levels in men, but not women, in the population-based Heinz Nixdorf Recall cohort study [36]. The latter study is of particular interest as it controlled for a large number of confounding variables. Nevertheless, the study produced some unexpected results, showing no effect modification by medication use or co-morbidities on the association between PM and fibrinogen.

*In summary, much of the recent epidemiological research has strengthened the evidence of associations between short- and long-term particulate air pollution exposure and changes in haemostasis. Increased use of assays of global coagulation, reflecting events from beginning of clot formation to fibrinolysis, has contributed to strengthening the evidence that particulate pollutants could cause thrombotic events. However, changes have been modest and, at times, inconsistent. Furthermore, it is not clear whether differences in susceptibility exist within populations (for example, age, sex, pre-existing disease). The magnitude of these changes is in general small, and their clinical relevance has yet to be ascertained. It is, however, possible that, if untreated, these haemostatic changes over the long-term could ultimately lead to exacerbation of myocardial ischaemia and other clinical outcomes in response to a triggering factor. The current evidence is too sparse to draw conclusions about the effects of exposure of gaseous pollutants on haemostasis.*

## Controlled human exposure studies

Controlled human exposure studies provide a means to investigate biological mechanisms for pollutants in isolation with fewer confounding variables. Despite the heterogeneity in study design and the possibility of publication bias against negative findings, it is difficult to deny that particulate air pollution promotes a pro-thrombotic state. In recent years, much of the research has focussed on DE and CAPs. A series of controlled human exposure studies have demonstrated impaired fibrinolysis, by measurement of t-PA (the activator of fibrinolysis) in healthy volunteers after exposure to DE generated under either transient engine speed and load (300 μg/m^3^; 1 h) [47] and idling (250 μg/m^3^; 1 h) conditions [48]. Similar observations have been observed in patients with coronary heart disease (CHD) following DE exposure [49] and in healthy volunteers after exposure to coarse CAPs (89.0 μg/m^3^; 2 h) [50].

In recent years, major advances have been made in supporting thrombus formation following DE exposure. Ex vivo thrombus formation has been assessed, using a Badimon chamber (mimics flow conditions within the coronary circulation of man), after DE exposures (320–350 μg/m^3^) lasting 1 and 2 h in healthy volunteers [51, 52]. Interestingly, DE exposure was associated with increased expression of platelet-leukocyte aggregates, as well as increased circulating levels of soluble forms of CD40L, suggesting that the enhanced thrombus formation was mediated through platelet activation [51, 52]. DE exposures did not have any significant effect on cellular and soluble markers of inflammation [51, 52]. The latter suggest that short-term exposure to DE may lead to a pro-thrombotic state, independent of systematic inflammation. However, these studies have been typically limited to measuring a small number of blood markers of inflammation, and cannot exclude other markers of inflammation. The question as to whether particulate air pollutants affect platelets through direct and/or indirect effects thus remains undetermined.

Significant advancements have been made in recent years as to whether the anti-fibrinolytic, and pro-thrombotic responses are due to diesel PM or the associated DE gaseous components (or both). Acute exposures of healthy volunteers to DE with a particle trap abolished the effects on endogenous fibrinolysis and ex vivo thrombus formation [51]. While the filters were effective in terms of particle removal (reduced particle concentration by 98%), the catalytic oxide coatings led to measurable increases in concentrations of NO_2_. Nevertheless, a study by the same investigators found that bradykinin-induced release of tPA into plasma did not change significantly after exposure to NO_2_ at 4 ppm for 1 h [53], suggesting that NO_2_ does not appear to be a major factor in the anti-fibrinolytic effects of dilute DE inhalation. Strengthening the role of particles, thrombotic effects appear to be relatively consistent in inhalation studies performed using diesel engine emissions at equivalent particle mass concentrations but different gaseous components [52]. However, controlled exposure to O_3_ (0.3 ppm for 2 h) reduced plasma PAI-1 within 24 h in healthy volunteers, suggesting a pro-thrombotic effect of O_3_ [54].

The source and chemical nature of the particles appears to be important. For example, exposure to CAPs, from non-exhaust traffic related sources, did not significantly affect the fibrinolytic balance in patients with CHD [55]. Furthermore, a pure carbon nanoparticulate exposure alone had no discernible effect on blood coagulation and fibrinolysis in healthy volunteers [56]. However, to date these effects have not been studied extensively and therefore only limited information is available on these issues. Although diesel motor emissions constitute a significant proportion of UFPs in the urban environment, it is as yet unclear which fraction of particulate from DE plays the largest role in the thrombolytic/fibrinolytic effects. Studies using inhaled laboratory generated ultrafine carbon particles (50 μg/m^3^; count medium diameter, 32 nm; 2 h) – as surrogates for ambient UFP- have reported platelet activation and increased vWF (mediates platelet-platelet or platelet-vessel interaction) levels in persons with type 2 diabetes [10, 57, 58].

Gene expression profiling analysis using microarray has demonstrated changes in expression levels of genes involved in the clotting cascade [59]. Expression of the F2R gene (located at chromosome 5q13.3), which encodes the thrombin receptor, were elevated 30-fold in 14 healthy participants after a 1 h exposure to diluted DE (300 μg/m^3^) compared to clean air [59]. In addition, DE exposure decreased expression of the gene encoding the urokinase-type plasminogen activator (PLAU), a secreted serine protease involved in the breakdown of clots [59]. Genes related to oxidative stress pathways have been shown to be differentially regulated in response to exposure to air pollution. In particular, inducible nitric oxide synthase 2A (NOS2A), a biomarker of oxidative stress, was upregulated in healthy volunteers 24 h after DE exposure (300 μg/m^3^, 60 min on 2 separate days) [59]. Further evidence is required to confirm a role for oxidative stress in causing PM-induced pro-coagulant/thrombotic effects. It is also worth noting that the changes in the gene expression level of coagulation markers have not been frequently reflected at the protein level after DE exposure.

*Taken as a whole, controlled exposure studies in man support the notion that particulate air pollution exposure favours a pro-thrombotic state. The most common human controlled inhalation studies are to DE. Exposures are acute (1–2 h) and concentrations used are high, but within the range that could occur in the urban environment (for example, during high air pollution episodes or in areas of dense traffic). It has been reported that O*_*3*_ *exposure, but not NO*_*2*_ *, promotes thrombosis, but these are isolated studies. A number of different mechanisms have been postulated to mediate thrombogenic actions of particulate air pollution. Arguably, one of the strongest hypotheses for the enhanced thrombotic profile in response to exposure suggests an increase in platelet activation. Inflammation and oxidative stress continue to be implicated as a mechanism underlying pollution-induced pro-thrombotic and anti-fibrinolytic effects, although findings are inconsistent. Most investigations used young, healthy subjects and there is insufficient data to ascertain whether pre-existing conditions further promote the thrombogenic effects of air pollution.*

## In vivo studies in animals

Animal models provide greater flexibility to explore the biological mechanisms of air pollutant exposure. In relation to the haemostatic system in particular, in vivo models in which induction of thrombosis can be directly studied, as opposed to relying on surrogates or biomarkers alone, or blood clotting ex vivo in the absence of the vessel wall, can be studied. Together with biomarkers, a detailed assessment of effect and mechanism is possible. In recent years, a variety of biological pathways have been argued persuasively. In terms of environmental sources of air pollution, the focus has remained on PM, through inhalation of urban ambient particles or instillation techniques to deliver particulates in the absence of gases. There is increasing evidence that the association between exposure to PM and alterations in haemostasis is mediated, at least in part, by interleukin-6 (IL-6). Mutlu et al. (2007) first drew our attention to a role of IL-6 in mediating PM-related pro-thrombotic effects [60]. In C57 mice, intratracheal administration of coarse ambient particles (PM_10_; 10 μg), collected from an urban background site in Düsseldorf, Germany, increased lung production of IL-6, reduced clotting times and enhanced thrombin generated within 24 h compared with saline instillation [60]. Additionally, the role of alveolar macrophages as the critical source of this elevated IL-6 was confirmed by depleting alveolar macrophages using intracheally instilled liposomal clodronate [60]. IL-6 dependent activation of coagulation has also been suggested in studies using different particle size fractions and other exposure methods [61]. Work by Budinger et al. (2011) demonstrated an IL-6-dependent increase in coagulation activation markers (thrombin-anti-thrombin (TAT)) following inhalation exposure (8 h per day, for 3 days) to fine CAPs (PM_2.5_; 88.5 ± 13.4 μg/m^3^) and after the instillation PM_2.5_ (200 μg) [61]. Unfortunately, because studies have used different methods in assessing the haemostatic effects, makes comparisons between studies difficult.

Budinger et al. (2011) also showed a role of IL-6 in the increased levels of TF antigen following PM exposure [61]. IL-6 knockout mice did not display the increased levels of TF following inhalation exposure (8 h per day, for 3 days) to fine CAPs (PM_2.5_; 88.5 ± 13.4 μg/m^3^) and after the instillation PM_2.5_ (200 μg) [61]. However, measurements of TF antigen do not necessarily reflect the functional capacity and integrity of TF [62]. Perhaps the most convincing evidence for a role of TF in mediating thrombus formation following exposure to PM comes from the study by Kilinç et al. (2011) [62]. In mice intratracheal administration of UFPs (approx. 0.36 μg) collected near a Dutch roadside tunnel (mainly used by heavy diesel trucks) increased plasma thrombin generation at 4 and 24 h post-exposure [62]. The extrinsic pathway is initiated when TF comes in contact with and activates factor VII (Fig. 3). Kilinç et al. (2011) showed that the increased thrombin generation 4 h after exposure to UFP to be blocked by administration of TF/FVIIa inhibitor [62]. TF-driven thrombin generation was supported by observations that thrombin generation parameters were similar in wildtype and FXII (important protein involved in the initiation of the intrinsic pathway; Fig. 3) deficient mice 4 h after UFP exposure [62]. Of note, this study did not provide evidence for a causal link between IL-6 and TF. The use of FXII knockout mice to study the role of the intrinsic coagulation pathway provided support for the intrinsic pathway in the later phases of thrombin generation (20 h after UFP exposure) [62]. While intratracheal instillation of UFP increased thrombin generation in wildtype mouse plasma at 20 h post-exposure, no effect was seen in FXII knockout mice. Similar results were obtained by pharmacological inhibition of FXII by the inhibitor corn trypsin inhibitor [62]. Overall, these results suggest distinct mechanisms regulate pollution-related haemostatic effects over different timescales. However, more studies are needed with air pollution from different sources and different particle sizes. Inhalation studies are also required. Intratracheal administration is a more artificial route of delivering pollutants to the lungs and does not simulate normal animal inhalation exposure conditions [63]. Nevertheless, intratracheal instillation of particle suspensions has been shown to be a reliable way of producing excellent dispersion of particles throughout the lobes of rodent lungs and across the surface of the alveoli, leading to pulmonary effects that are directly comparable to that of inhalation studies [64, 65].

The study by Kilinç et al. (2011) did not assess whether the initiation of the intrinsic coagulation pathway following exposure was a consequence of an inflammatory response and/or particle translocation processes [62]. Interestingly, Budinger et al. (2011) suggested that IL-6 is significantly associated with PM-induced thrombogenic effects independent of other inflammatory markers [61]. Two studies have been published examining the effect of anti-inflammatory agents on thrombogenic factors in mice following exposure to DEP (15–30 μg) [66, 67]. Both studies demonstrated a critical role for inflammation in mediating DEP-induced thrombotic effects [66, 67]. Interestingly, the Nemmar et al. (2003) found that inflammation and thrombosis were associated events at 18 h, but not at 4 h [68]. Particle translocation could play a role in the early pro-thrombotic effects of DEP, with inflammation playing a greater role at later stages. Indirect evidence for this concept is provided by the finding that intravenous administration of DEP to the blood has the capacity to increase in vivo thrombosis formation at 2 h, without inducing inflammation [69]. The link between inflammation and thrombosis at later time points after pulmonary instillation is possible (6–24 h), but complex [69]. Smyth et al. (2017) showed intratracheally instillation of DEP (25 μg) in mice to induce platelet aggregation independent of lung inflammation [70]. The study also showed that platelet aggregation persisted in endothelial nitric oxide synthase (eNOS) knockout mice [70], suggesting a lesser influence of vascular-derived mediators in actions of DEP on platelets. There is some discrepancy regarding the role of platelets in mediating the pro-thrombotic effects of particulate air pollution [13, 51, 52, 60–62, 69, 70]. This is likely due to differences in study designs, including species, particle types, doses, exposure methods and different measurable indicators of platelet function. An especially noteworthy study is that by Emmerects et al. (2012) suggesting that continuous exposure of mice to traffic-related air pollution, in a real-life setting (mice were placed in a highway tunnel for 25 or 26 days; mean 24.9 μg/m^3^ PM_2.5_) may affect platelet function with an increased release of platelet derived pro-coagulant microvesicles [13]. This study also looked at how age modifies the pollution-induced changes in platelet counts and activation but no clear patterns emerged [13]. More studies assessing the potential effect modification by age, as well effects of co-morbidities are clearly needed. One study has examined hypertension and demonstrated enhanced platelet aggregation and thrombin generation 24 h after intratracheal instillation of DEP (15 μg) in mice induced with experimental hypertension compared with wildtype controls [71]. There is limited evidence to support the assertion that exposure to air pollution may have a priming effect that leads to an augmented response to subsequent stimuli [72]. Platelets from CAPs-exposed mice (PM_2.5_; 88.5 μg/m^3^; 6 h/day; 5 days/week for 2 weeks) showed a 54% increase in fibrinogen binding in response to the agonist adenosine diphosphate (ADP), compared to saline exposed mice [72].

As discussed above, impairment of endogenous fibrinolysis has been suggested from studies exposing humans to DE by inhalation under controlled experimental conditions [47, 49]. Subsequent studies showed that this effect was directly attributable to the exhaust particles [73]. Increased aorta PAI-1mRNA levels, suggesting the presence of vascular injury and/or fibrinolytic activation were observed in rats exposed to O_3_ (0.38 ppm) or DEP (2.2 mg/m^3^) alone for 16 weeks (5 h/day, 1 day/week) [73]. Interestingly, aortic thrombodulin (TM) and t-PA mRNA levels also increased, which are opposite to the expected direction of change [73]. Further studies are needed to determine whether these exposures would reflect similar changes in corresponding protein levels or activity. A great advantage of the study design employed by Kodavanti et al. (2011) was that they analysed disparities in exposure to both acute (2 days) and sub-chronic (16 weeks) pollution in the same study. The analysis found that the associations between exposure and mRNA markers of haemostasis to be strongest after a longer series of exposures [73]. This study also showed a synergistic decrease in effects following DE and O_3_ (0.5 ppm + 2 mg/m^3^ 5 h/day; 1 day/week for 16 weeks) co-exposure [73]. Concentrations of PAI-1 mRNA in mouse fat tissue has also been shown to increase following inhalation exposure (8 h per day, for 3 days) to fine CAPs (PM_2.5_; 88.5 ± 13.4 μg/m^3^) and after the instillation PM_2.5_ (200 μg) [61]. Notably, studies in IL-6 null mice revealed no significant effect of the loss of IL-6 on the induction of PAI-1 expressions following exposure [61]. Treating wildtype mice with the TNF-α receptor antagonist etanercept (10 mg/kg i.p.) prevented upregulation of PAI-1 expression following exposure [61]. In contrast etanercept had no effect on PM-induced thrombus formation [61]. Another study showed curcumin (45 mg/kg), a compound with anti-inflammatory and anti-oxidant properties, to block the DEP-induced up-regulation of TNF-α and PAI-1 in mice (15 μg DEP via intratracheal instillation, 4 times/week, for 1 week), but to only partially inhibit PM-induced thrombosis [66]. Collectively, these data suggests that PM exposure is associated with both the activation of coagulation and impairment of fibrinolysis, but that these facets are regulated via distinct mechanisms.

Lastly, this review found limited evidence linking oxidative stress to the effects of pollution exposure on thrombosis and fibrinolysis. For example, the pulmonary and systematic inflammation induced by ultrafine carbon particles (180 μg/m^3^, 24 h) in aged spontaneously hypertensive rats (SHRs) was associated with increased hemeoxygenase-1 levels (HO-1; a marker of oxidative stress), alongside changes in biomarkers of thrombosis [74]. Additionally, oxidation of the low density lipoprotein (LDL) receptor has an influence on the thrombotic effects of inhaled vehicular emissions [73].

*In summary, the majority of animal studies support the notion that particulate air pollution exposure leads to an enhanced thrombogenicity. The use of* in vivo *of thrombus formation in blood vessels* in situ *is a strength of animal studies given that clot formation will be heavily influenced by the vascular wall and flow conditions of the blood prior to clot formation. Whilst* in vivo *studies generally use high air pollution exposures, the similarity of many of the mechanistic pathways with those shown in epidemiological and controlled exposure studies in man suggests that these exposures are relevant. Multiple mechanisms have been postulated, including inflammation, oxidative stress, interplay between TF and IL-6 (potentially independently of other inflammation pathways), increases in coagulation factors, and impaired fibrinolysis. A role of platelet activation in the enhanced thrombosis is one of the most consistent observations. The thrombotic effects of gaseous pollutants and the use of models of susceptible populations is an avenue for future research, and one that would provide a useful foundation addressing these matters in human studies, particularly if dose and time responses can be defined.*

## Summary and conclusions

In 2015, ambient air pollution was estimated to be the 5th leading risk factor for death globally, with CVD accounting for the majority of these deaths [2]. Given the global commitment to reduce premature non-communicable diseases by 25% by 2025 [75], an improved understanding of the mechanisms underlying the significant detrimental effects of air pollution on CVD will help achieve this target. Specifically, improved knowledge of the mechanistic pathways linking air pollution to negative effects on cardiovascular health has the potential to improve policy, and ultimately improve health and life expectancy of people by allowing target intervention. In 2010, a seminar review of literature suggested a role of the haemostatic system to the overall cardiovascular effects of air pollution, although a great deal of uncertainty remained [4]. This review discusses the growing body of evidence from 2009 to 2016, expanding the remit to include gaseous co-pollutants as well as airborne particulates of different size fractions.

A large body of work (2326 references screened, 74 assessed) has been published on the topic in the last seven years. Overall, examination of this literature supports the contention that exposure to air pollution promotes coagulation and impairs fibrinolysis, leading to an unfavourable imbalance in haemostatic factors that would be expected to increase the risk of thrombotic events in susceptible individuals. As noted in the AHA review, inconsistent findings between studies are commonplace. However, the volume of publications supporting the pro-thrombotic effect of air pollution far outweigh those showing no effect or the opposite effect, even given the assumption that there may be a publication bias towards studies suggesting a health risk of pollutions. Potential reasons for the inconsistencies are many. In general, the investigations assessed were, in our opinion, of high scientific quality – both in their design, implementation and analysis. Instead, a major source of discrepancies most likely reflects the differences in study design and increasing complexity of the endpoints open for investigation. Additionally, the nature of exposure is also likely to play a significant role in the differences between studies, in a manner that will depend on the study type under investigation. For example, distinguishing the effects of individual pollutants remains a challenge for epidemiological studies, especially where there is high correlation between pollutants (e.g. PM_2.5_ and NO_2_ from traffic-derived sources). Controlled exposure studies in man and animals are frequently criticised for the high concentrations of their exposures; a criticism that still applies to many of the studies performed in recent years. Indeed, little consensus has been reached in striking a balance between using a realistic dose that models long-term exposure with obtaining suitably high exposure to explore pathogenic mechanisms in short-term studies. However, the number of studies, both epidemiological and experimental, assessing the effects of longer periods of exposure are increasing. Overall, results of these studies are somewhat reassuring in that the direction of effect matches those of short-term studies.

While the remit of this literature review was to address whether gaseous co-pollutants have haemostatic effects, there have been too few studies to make this assumption with any certainty. Epidemiological studies suggest that it is likely that ambient levels of gases such as NOx, O_3_, and possibly SO_2_, are associated with biomarkers of thrombotic pathways, however, a paucity of experimental studies means it is not possible to draw any conclusions as to the wider functional consequences of such observations, or address more taxing issues such as causality. Of those few studies available, it does appear that gases can alter thrombotic pathways, however, initial indications suggest that inconsistences between studies could be even greater than that of PM; an observation that might not be especially surprising given that our focus is on a physiological system (the blood) some distance from the initial organ of exposure (the lung). Obtaining evidence of a dose-dependency of the effects of gases will again be important for advancing this area.

**Fig. 5:**
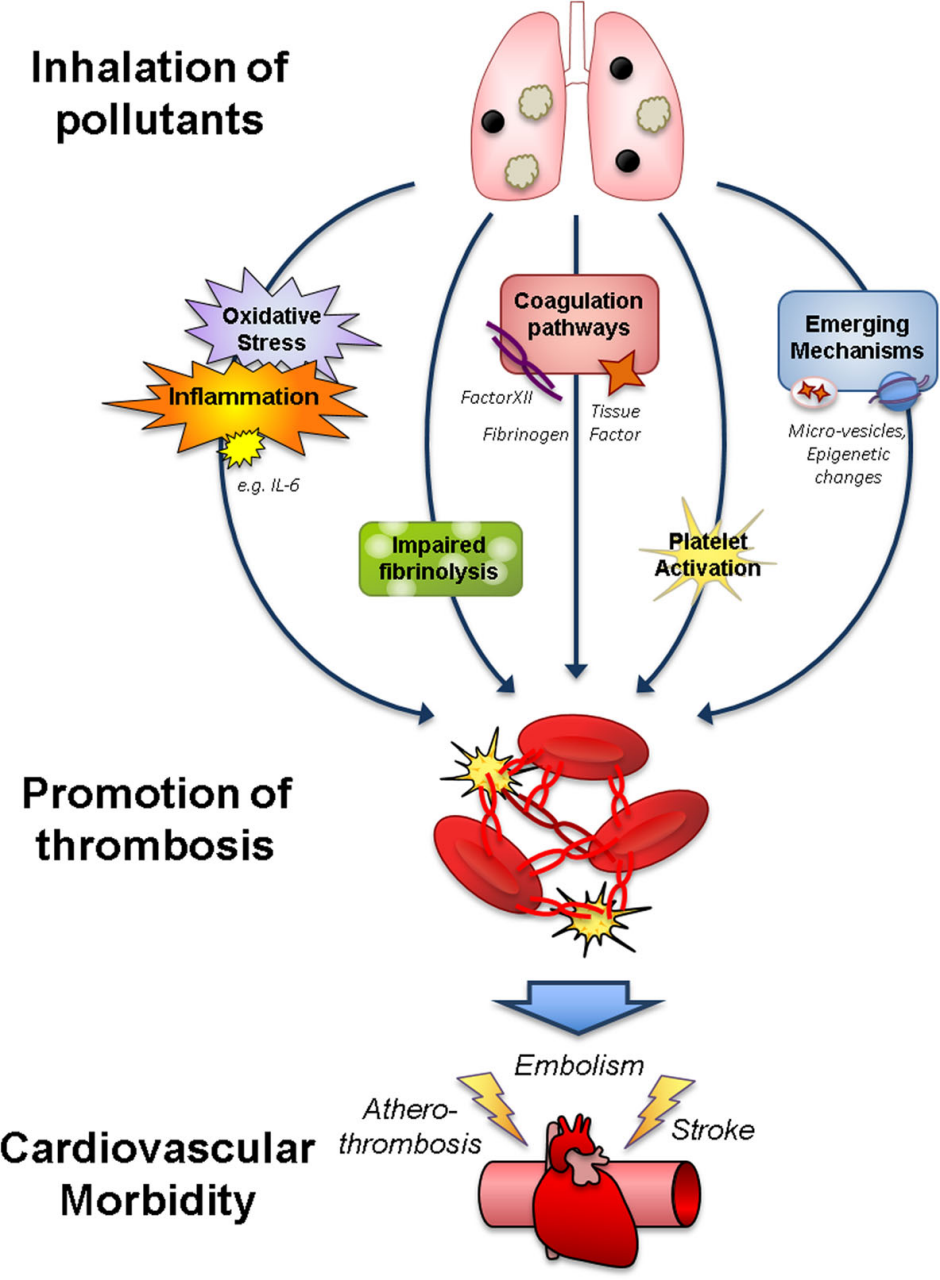
Summary of the main and emerging mechanisms described in the review

While our understanding of the pathophysiologic mechanisms by which air pollution promotes thrombosis remains incomplete, significant progress has been made. Similarities in the pattern of biological actions on the haemostatic system suggest that the findings of short-terms studies provide relevant, and valuable, information. Multiple mechanistic pathways are plausible, including platelet activation, oxidative stress, interactions between inflammatory mediators and impaired fibrinolysis. Emerging pathways of interest include the role of circulating microvesicles, epigenetic modifications and alterations in sensitivity to the above caused by genetic polymorphisms. A schematic overview of these pathways is shown in Fig. 5. There is great complexity in the coagulation cascade and its interaction with other pathways leading to thrombosis (see Figs. 2 and 3), with many levels of feedback regulation (positive and negative). Subsequently, it has been difficult to disentangle which points in the pathway are pivotal in driving the haemostatic effects of PM. Increases in circulating fibrinogen are one of the more consistent observations across studies, thus the availability or activation of this mediator could be significant. In the same respect, platelets (and platelet-derived mediators) appear to play an important role in driving the pro-thrombotic effects of air pollution. It is likely that these mechanisms act in concert, and potentially acting synergistically to amplify the overall effect on the blood. The mechanism by which inhaled pollutants cause thrombotic effects in distance vascular beds has not been elucidated further to any great degree in the publications reviewed here (refer to [19, 20, 76, 77] for this), although inflammatory and oxidative mediators remain a possibility, and building evidence for the translocation of nano-sized particles into the circulation offer the potential that these particles could directly interfere with blood factors. Experimental studies using interventions (e.g. pharmacological inhibition, genetic modifications) to inhibit specific pathways will be useful in dissecting out the key pathways of interest. Ultimately reducing the health effects of air pollution will rely on removal of pollutants and a better understanding of these mechanisms will help ascertain which pollutants are most harmful and which populations are most at risk. Additionally, while primary prevention is the main desire in this field, there could be value in identifying medications to prevent specific thrombotic pathways for those individuals who may be especially susceptible (e.g. patients with a prior history of ischaemic stroke or CAD) that have an unavoidable exposure to raised levels of air pollution (e.g. reside close to traffic).

In summary, the recent evidence supports a role for a pro-thrombotic effect of air pollution, through the activation of multiple pathophysiological processes. It is highly likely that these effects will contribute to the overall cardiovascular morbidity associated with air pollution and increase the risk of thrombotic effects in those with pre-existing CVD. A clearer understanding of dose-dependency of effects and of the effects of longer-term exposures, would greatly add to the case for causality of the associations between long-term average ambient concentrations of air pollutants and indices of cardiovascular morbidity. Key area of future research will be to assess the role of gaseous pollutants and studies that directly compare potentially susceptible individuals/models with healthy counterparts.

## Abbreviations

ADP: Agonist adenosine diphosphate
AHA: American Heart Association
APTT: Activated partial thromboplastin time
CAD: Coronary artery disease
CAP: Concentrated ambient particles
CB: Carbon black
CHD: Coronary heart disease
CO: Carbon monoxide
COPD: Chronic obstructive pulmonary disease
CRP: C reactive protein
CVD: Cardiovascular disease
DE: Diesel exhaust
DEP: Diesel exhaust particles
eNOS: endothelial nitric oxide synthanse
ETP: Endogenous thrombin potential
FVII: Factor VII
H: hour
HO-1: Hemoxygenase-1
IL-6: Interleukin-6
IQR: Interquartile range
Kg: kilogram
LDL: Low density lipoprotein
mg: milligram
NO_2_: Nitrogen dioxide
NOS 2A: Nitric oxide synthase 2A (NOS2A)
NOx: Nitric oxides
O_3_: ozone
PAI-1: Plasminogen activator inhibitor-1
PLAU: urokinase-type plasminogen activator gene
PNC: Particle number concentration
Ppm: parts per million
PT: Prothrombin time
sCD40L: soluble CD40 ligand
SES: Socio-economic status
SHRs: Spontaneously hypertensive rats
SO_2_: sulphur dioxide
sP-selectin: soluble platelet selectin
TAT: Thrombin-anti-thrombin
TF: Tissue factor
TM: Thrombodulin
TNF-α: Tumour necrosis factor-alpha
TPA: Tissue plasminogen activator
UFP: Ultrafine particle
vWF: von Willebrand factor
μg: microgram
